# Knowledge and Confidence of Physician Assistant Students in Managing Patients with a Documented Penicillin Allergy

**DOI:** 10.3390/antibiotics15010094

**Published:** 2026-01-16

**Authors:** Kayla Moody, David Weil, Sarah Jane O’Neal, Nicole Sunshine, P. Brandon Bookstaver

**Affiliations:** 1 Duke University Hospital, 2301 Erwin Rd., Durham, NC 27710, USA; 2 Department of PA Studies, Wingate University PA Program, 515 N. Main St., Wingate, NC 28174, USA; 3 Department of PA Studies, Wake Forest University School of Medicine, 110 N. Pinnacle Ridge Rd., Beech Mountain, NC 28604, USA; 4 Department of Clinical Pharmacy & Outcomes Sciences, College of Pharmacy, University of South Carolina, 715 Sumter Street, Columbia, SC 29208, USA

**Keywords:** allergy, penicillin, beta-lactam, physician assistant, health professional education

## Abstract

**Objective:** Physician assistants (PAs) are frequently involved in managing acute bacterial infections in patients with documented penicillin (PCN) allergies. Inappropriate antibiotic choice in patients with existing allergies may place them at undue risk. This study aimed to assess the knowledge and confidence among PA students in managing patients with documented PCN allergies. **Methods:** An electronic survey was distributed to enrolled students in participating PA programs in North and South Carolina. The survey tool consisted of 20 questions with 13 focused on knowledge and confidence primarily scored on a 5-point Likert scale. Data were collected and protected via the REDCap^®^ database. Primary objectives were knowledge of penicillin allergies and confidence in management decisions. Sufficient knowledge was considered a score of 80% or greater; adequate knowledge was considered 70% or greater on relevant assessments. **Results:** Overall, 406 students from 10 unique programs completed the survey. They were predominantly female (76%) with 43% in the first year of their program. The mean student knowledge score was 25.9%, and 30% of respondents achieved adequate knowledge. Respondents reported an average cross reactivity between penicillin and beta-lactams of 29% (10–63%), cefazolin 50% (24–75), ceftriaxone 29% (11–60), and carbapenems 26% (8–50). The majority of respondents (66.5%) reported high levels of confidence in managing patients with penicillin allergies. **Conclusions:** The study found significant discordance between PA students’ high level of confidence in assessing patients with a PCN allergy and their comparative knowledge. PA students are likely to avoid beta-lactam antibiotics when there is a documented penicillin allergy, regardless of the documented reaction or low likelihood of cross-reactivity. Further training and education will help to encourage appropriate prescribing in these high-risk patients.

## 1. Introduction

Nearly 20 million adults in the United States report having experienced an allergic reaction to an antibiotic in the penicillin drug class [1]. However, the reactions reported are rarely clinically relevant; significant IgE-mediated or severe Type IV reactions are estimated to occur in fewer than 1% of all cases [1].

Reconciling penicillin allergies is an important stewardship tool to optimize antimicrobial decision-making and is a shared interprofessional initiative. Prescribing broad-spectrum antibiotics or second-line options is often more common among patients with unverified penicillin allergies. The use of alternative, non-preferred antibiotic options in these allergic patients contributes to antimicrobial resistance and is also associated with worse clinical outcomes and increased adverse events, including increased risk of surgical site infections (SSI) and *Clostridioides difficile* infections (CDI) [2,3,4]. Mechanisms to improve allergy reconciliation include interprofessional education initiatives on allergy history; cross-reactivity among beta-lactam antibiotics, expanded access to oral antibiotic challenges; and penicillin skin tests [5,6].

While clinicians often agree that penicillin allergy reconciliation and de-labeling in the electronic health record are critically important, their confidence and knowledge in managing these patients may be low or discordant [7,8,9]. Previous studies have shown knowledge gaps among healthcare providers and clinicians in the interpretation of beta-lactam cross-reactivity and related concepts in patients with a reported penicillin allergy [8,9] Given the frequency of encountering these patients, allergy reconciliation, knowledge of cross-reactivity, and risks of documented allergies should be emphasized in health science student training. Data from several assessments among pharmacy students and faculty suggest there may be deficits in knowledge of management of patients with documented penicillin allergies and related stewardship concepts [10,11,12]. To date, little is known about knowledge and confidence among other health science students in managing patients with a penicillin allergy. Given that PAs work in numerous settings where antibiotics are frequently prescribed, it is critical to evaluate their training and understanding of antibiotic allergies [13].

The purpose of this study is to investigate knowledge of cross-reactivity among penicillin and other antibiotics and confidence in the ability to manage a patient with a documented penicillin allergy among PA students. 

## 2. Results

The total number of students enrolled was 940 (Table 1), and 406 (43%) completed the survey tool. Ten schools participated in the study and supplied, on average, 37 ± 29.05 students per program, with the minimum contribution being 15 students. Of those who completed the demographic section of the survey, 73.7% self-identified as female. Expected graduation year was recorded, with 43.4% of participants selecting 2022 (1st year), 34.7% selecting 2021 (2nd year), and 13.1% selecting 2020 (3rd year).

Among the participants who reported a likelihood of avoiding beta-lactam prescribing in various documented allergy scenarios, 63.8% indicated avoidance in patients with a history of nausea/vomiting, and 91.1% avoided prescribing in patients with documented previous facial swelling. Furthermore, 95.6% of respondents were likely to avoid this class of antibiotics in patients with a documented Stevens-Johnson Syndrome (SJS) diagnosis.

In questions evaluating the likelihood of cross-reactivity among penicillin and other beta-lactams, the median cross-reactivity estimate was 29% (10–63). Among the cephalosporins, participants’ median cross-reactivity estimates for ceftriaxone and cefazolin were 29% (11–60.3) and 50% (24–75), respectively. The estimated cross-reactivity with carbapenems demonstrated a median likelihood of 26% (8–50.3), while the estimated cross-reactivity with sulfamethoxazole/trimethoprim had a median of 16% (8–40.8). The average aggregate knowledge score among the entire population was 25.9%. Sixteen participants (4%) were included in the sufficient category by achieving a knowledge score greater than the 80% threshold (Table 2).

**Figure 1:**
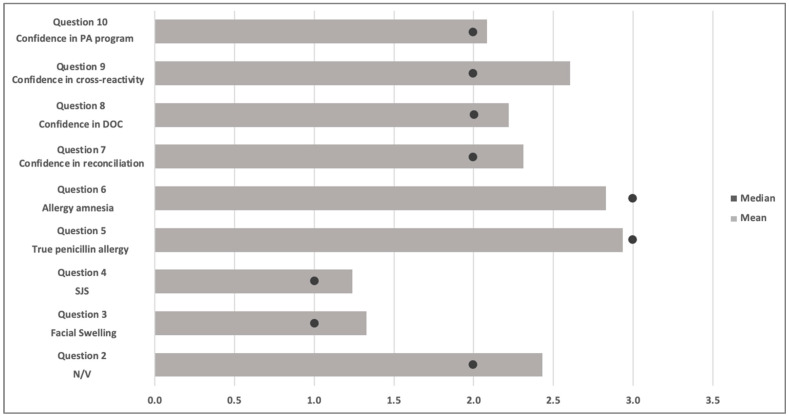
Likert Scale responses for Survey Questions 2–10 regarding confidence and likelihood of avoiding a penicillin. Note, the circle represents the median.

**Figure 2:**
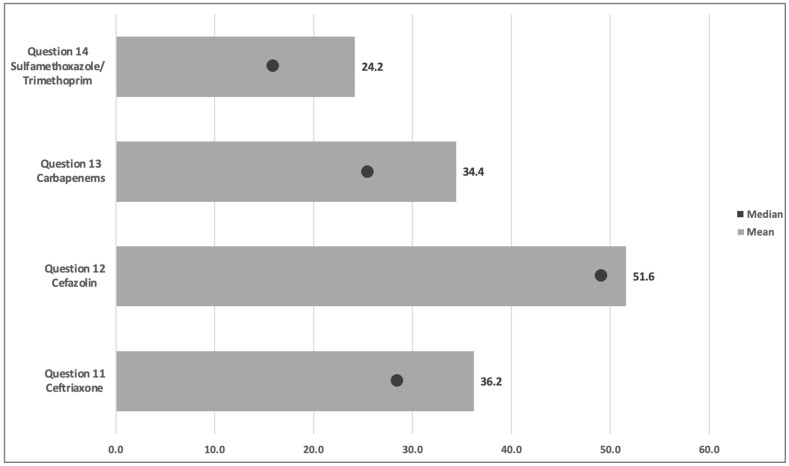
Responses for Likelihood (Percentage) of Cross-Reactivity between index drug and penicillin. Note, the circle represents the median.

Of the 398 participants included in the confidence analysis, 289 (66.5%) had an aggregate confidence score considered positive or affirming confidence in effectively managing penicillin-allergic patients. There was a significant difference in the proportion of students who answered correctly on several knowledge-based questions between the group that was not considered confident and the group that was. The confident group was significantly more likely to avoid beta-lactams in previous facial swelling (*p* = 0.02) and SJS (*p* = 0.03). Furthermore, respondents with high confidence were significantly more accurate across all cross-reactivity estimates, including ceftriaxone (*p* < 0.001), cefazolin (*p* < 0.001), carbapenems (*p* < 0.001), and sulfamethoxazole/trimethoprim (*p* < 0.001) (Figure 1 and Figure 2). Among respondents who self-reported as confident in managing penicillin-allergic patients, 18% (53) of individuals received an average score within the threshold for acceptable knowledge, compared to 9% (4) amongst students who did not report confidence in allergy management. There was no difference in sufficient knowledge; both groups had very low scores, with the overall cohort showing that only 4% of students (16) had knowledge scores greater than 80% (Table 3 and Table 4).

In comparing first-year and upper-level students, the latter recorded statistically higher confidence scores (*p* < 0.001). Likewise, upper-level students’ cross-reactivity estimates were significantly closer (*p* < 0.001). Furthermore, twice as many participants in the upper-level group (26% versus 13%) had an average knowledge score within the acceptable range.

Of the participants, 78% (319) had not received any training in managing patients with penicillin allergies outside of their PA program, and 73.7% (269) expressed interest in receiving additional formal training, such as a certificate program.

## 3. Discussion

This study is the first that we are aware of to evaluate PA students’ knowledge and confidence in managing penicillin-allergic patients. Our results show that the overall knowledge of penicillin allergies and the likelihood of cross-reactivity with other antibiotics are deficient. This was discordant with the high self-reported confidence in the ability and training of the students. Our results provide perspective regarding the need for further education and training in penicillin allergy education and management.

The beta-lactam class of antibiotics includes penicillins, cephalosporins, and carbapenems. These agents share the chemical structure of a beta-lactam ring, which incites concern for potential cross-reactivity. Aside from Type IV reactions, such as SJS, very few reactions warrant the complete avoidance of beta-lactams. The true incidence of cross-reactivity between the beta-lactams is generally very low, comparing penicillins to cephalosporins and carbapenems [14,15]. Therefore, most patients can safely tolerate beta-lactams with a low risk of reaction, even with a reported penicillin allergy. This contrasts with our findings that show avoidance of a cephalosporin or carbapenem in up to half of patients with a reported penicillin allergy. Our results also demonstrated a wide standard deviation, suggesting a general lack of understanding of the subject.

It has been shown that Type I (IgE-mediated) penicillin allergies wane over time and patients can become tolerant of penicillin agents. Multiple studies demonstrate low prevalence of position reactions to penicillin class skin and oral sensitivity testing in patients tested years after their documented reaction [16,17,18]. This contradicts the responses found in our survey, which provides insight into education regarding the history and future presentation of documented penicillin allergies. PA student surveys also found that participants unnecessarily avoided other beta-lactams in patients who reported nausea and vomiting from prior penicillin use. Gastrointestinal side effects are one of the most common side effects of antibiotics and do not represent a true allergy [19]. Additionally, a small percentage of the cohort recommended the addition of a beta-lactam in the presence of SJS, which should not be re-challenged after occurrence due to severity [20]. This gap between confidence and knowledge may result from outdated or oversimplified information from previous healthcare exposures or general biases and assumptions from non-expert sources. Overconfident providers may be less likely to adhere to patient safety guardrails, consult colleagues, seek new educational resources, and ask for supervision. This can result in inappropriate antibiotic prescribing, patient miseducation, and worse patient outcomes, including increased side effects and/or treatment failure from inadequate coverage of second-line options.

Unfortunately, this discordance is not exclusive to PA students. A similar study published by Brezis M et al. explored whether overconfidence relates to physician reluctance to admit to medical errors. This study was conducted on medical students and physicians using a clinical vignette of a female patient with a urinary tract infection and penicillin allergy. The form asked participants to rate their confidence in each step of diagnosis and management. They found high confidence levels regardless of accuracy, which was emphasized in the physician group. This overconfidence resulted in a reduced number of disclosed errors. The group concluded that there is a need for further emphasis on knowledge and adequate self-doubt in medical training [21]. Previous work by Kufel et al. evaluating faculty perception of pharmacy students’ capability to evaluate and manage penicillin allergies after curriculum completion showed great variability in allergy education across 138 US colleges of pharmacy. This study noted that faculty considered a high percentage of students unprepared to evaluate and manage penicillin allergies, although perception of student readiness was higher for students who had completed case-based education (54.5% vs. 25.0%, *p* < 0.01) [11]. Fundamental concepts, such as penicillin cross-reactivity, have been emphasized in core curricula for other health professional training, while some allergy management strategies (allergy interviewing, skin testing, oral challenges, etc.) are often a part of elective curricula instead [11]. This is reflected by Wagner et al., where a survey of pharmacy students demonstrated gaps in understanding and confidence on beta-lactam allergy assessment and intervention despite 84% of students reporting that the topic was present in their curriculum [12]. The authors of both studies emphasize that increased knowledge and confidence can be achieved with the integration of comprehensive didactic learning with case-based and lab-based simulations on allergy management. All PA programs are required to teach about antibiotics, with many doing so through a pharmacology course; however, the level and type of instruction is up to the PA program itself.

Physician assistants serve the healthcare needs of a significant portion of the public health sector. As of 2024, 22.0% of physician assistants work in primary care practices in the United States [22]. This role presents a unique opportunity to reconcile penicillin allergies and educate patients on the rarity of cross-reactivity. Therefore, creating opportunities to ensure PA comprehension of the topic is important to provide the most appropriate therapy available. One such opportunity is through continuous education (CE) programs, as outlined by Covington EW et al. In this study, researchers evaluated the knowledge gaps and comfort levels of pharmacists and pharmacy technicians in managing penicillin allergies. The results concluded that a 1 h targeted CE program increased overall knowledge on the subject and was maintained for up to 2 months after the presentation [9]. Such programs can aid in dispelling the discord of knowledge and confidence, as seen in our results. These programs could be implemented into curricula across healthcare professions and modeled on clinical rotations to improve the knowledge of caring for penicillin-allergic patients. Early implementation of this practice has yet to be evaluated; however, this study provides further evidence that education on penicillin allergy reconciliation can improve knowledge and, ultimately, patient outcomes.

Due to the nature of the study, certain limitations were deemed unavoidable. Firstly, all survey questions were hypothetical and could be misinterpreted. Next, the study included a large proportion of first-year students who may not have learned about allergy reconciliation or cross-reactivity in their curriculum yet. This may be explained partially by differences in program duration and the timing of clinical experiences, as some programs are 2 years and year-round compared to three-year programs. Moreover, this was only conducted in 10 PA programs across North Carolina and South Carolina. Variations in curriculum between schools of different program duration and geographical regions may narrow external validity. 

Future studies of this topic could include surveying a larger geographical region of PA schools to increase external validity. To avoid limitations of differences in curriculum timing and duration for allergy education, a survey of newly licensed practitioners could be done. Furthermore, a multidisciplinary survey of all healthcare students’ knowledge and confidence regarding the care of penicillin-allergic patients could aid in the understanding of educational opportunities and improved patient outcomes.

## 4. Methods

This cross-sectional, survey-based study was conducted among ten participating PA programs in South Carolina and North Carolina. The University of South Carolina was the sponsoring IRB and each study site received IRB approval as needed. An on-site faculty member served as the liaison to students in different years of the PA program. This ensured the appropriate delivery of the electronic survey via email to each student. The survey was distributed via a link to a data collection tool within the REDCap^®^ (Research Electronic Data Capture) database hosted at the University of South Carolina [23]. The survey tool (Supplemental Table S1) consisted of 20 questions, primarily scored on a Likert scale (1 = strongly agree, 5 = strongly disagree). Demographic questions were mostly reserved for the end of the survey to increase participation. There were three questions assessing attitudes regarding antimicrobial selection, six questions assessing knowledge of penicillin allergies and the likelihood of cross-reactivity, and four questions assessing confidence in identifying and managing a patient with a penicillin allergy. The survey was developed by a team of physician assistant program faculty and an infectious diseases pharmacist expert in allergy assessment and evaluation to assess the expected knowledge of physician assistants. This tool was piloted on a group of analogous health professional students from the nursing and pharmacy programs, with feedback and revision resulting in no major changes. Participants needed to answer a minimum of 33% of the questions to be included. Participating students could enter a random draw to win one of six Amazon^®^ gift cards valued at $50 each. A separate voluntary survey link was provided to participants for the draw to maintain the anonymity of the survey results and prevent identifiable information from being included/accessible. The survey remained open for 6 weeks and students were sent reminder emails requesting their participation every 2 weeks.

The primary objective of this study was to identify PA students’ knowledge and confidence in managing patients with penicillin allergies. Knowledge was assessed as an aggregate score of the case scenarios and the questions on cross-reactivity among beta-lactams. A score above 80 percent was considered proficient, and a score of less than 80 percent was considered deficient. Acceptable knowledge was defined as a score between 70 and 79 percent from existing PA curriculum benchmarking of 70 percent as basic competency. These cut-offs were developed using the ACGME Milestones for development based on the Dreyfus model [24]. A median (IQR) and mean (SD) were calculated for individual and aggregate knowledge data. The proportion of participants with deficient scores and/or incorrect responses was determined. Then, a comparison between respondents with sufficient and deficient knowledge was conducted.

In addition, respondents with high and low levels of confidence were also assessed. A median (IQR) and mean (SD) were calculated for the responses to Likert scale questions assessing attitude and confidence. Responses of “agree” or “strongly agree” were considered positive or affirming of confidence, while responses of “neutral,” “disagree,” or “strongly disagree” were considered negative or demonstrating a lack of confidence. A comparison between those with high levels of confidence and low levels of confidence was performed. Additionally, survey responses were compared between first-year students and upper-level students. Categorical variables were analyzed using a Fisher’s exact or chi-square test as appropriate, and continuous variables were analyzed using a student’s *t*-test or Mann-Whitney U test. 

## 5. Conclusions

This study found a disconnect in the knowledge and confidence surrounding penicillin allergies, leading to incorrect clinical decisions among PA students. Inadequate antimicrobial stewardship and provider overconfidence or even discomfort with penicillin allergies can lead to avoiding first-line beta-lactam antibiotics, which has a strong association with poor patient outcomes. Therefore, there is a clear need to address penicillin allergy education within PA curricula.

These opportunities for curricular improvement can be included in didactic and experiential learning with encouragement for lecturers and preceptors to emphasize effective assessment and management. There are a variety of penicillin allergy assessment and management professional training, continuing education, and national certification programs that would benefit students. In a competitive employment environment, including education and/or certification in penicillin-allergy management in core curricula would provide students with a unique, marketable skillset as they transition to the workplace. By providing students with didactic, case-based, and hands-on training, physician assistants will be empowered to enhance patient outcomes through improved antibiotic prescribing, allergy de-labeling, and participation in antimicrobial stewardship initiatives.

## Figures and Tables

**Table 1:** Demographics *.

Question	N (%)
**Gender**	
Female	299 (73.7%)
Male	62 (15.3%)
**Anticipated graduation year**	
Grad 2020 (3rd year)	53 (13.1%)
Grad 2021 (2nd year)	141 (34.7%)
Grad 2022 (1st year)	176 (43.4%)
**I have received training in managing patients with penicillin allergies outside of my PA program.**	
Yes	46 (11.3%)
No	319 (78.6%)
**I or someone in my immediate family has a documented penicillin allergy.**	
Yes	96 (23.7%)
No	273 (67.2%)
Prefer not to answer	2 (0.5%)

* Where percents do not total 100%, the remaining percentage is a question not answered by respondent.

**Table 2:** Summary of Confidence and Knowledge.

Confidence in One’s Own Ability to Manage Patients with Penicillin Allergies	N (%)
Students with positive aggregate confidence scores	289 (66.5%)
**Student score on knowledge section** **(≥80% = “sufficient knowledge”, 70–79% = “acceptable knowledge”)**	**(%)**
Average Score (entire cohort)	25.9%
Students who achieved “sufficient knowledge” (entire cohort)	4%
Final year students who achieved “acceptable knowledge”	26%

**Table 3:** Confidence and Attitudes *.

Question	N (%)
**I am confident in my ability to assess/reconcile a patient’s documented penicillin allergy.**	
Strongly Agree/Agree	270 (66.5%)
Neutral	62 (15.3%)
Strongly Disagree/Disagree	66 (16.3%)
**I am confident in determining the drug of choice for patients with a documented penicillin allergy.**	
Strongly Agree/Agree	292 (71.9%)
Neutral	43 (10.6%)
Strongly Disagree/Disagree	63 (15.5%)
**I am confident in my knowledge of cross-reactivity among other antibiotics in patients with a documented penicillin allergy.**	
Strongly Agree/Agree	232 (57.1%)
Neutral	60 (14.8%)
Strongly Disagree/Disagree	106 (11.1%)
**I feel confident in my training during the PA program in managing patients with penicillin allergies.**	
Strongly Agree/Agree	295 (72.75)
Neutral	58 (14.3%)
Strongly Disagree/Disagree	45 (11.1%)
**How likely are you to avoid a beta-lactam antibiotic in a patient who reports an allergy to penicillin with nausea and vomiting?**	
Very Likely/Likely	259 (63.8%)
Neutral	44 (10.8%)
Very Unlikely/Unlikely	87 (21.4%)
**How likely are you to avoid a beta-lactam antibiotic in a patient who reports an allergy to penicillin with facial swelling?**	
Very Likely/Likely	370 (91.1%)
Neutral	4 (0.9%)
Very Unlikely/Unlikely	21 (5.2%)
**How likely are you to avoid a beta-lactam antibiotic in a patient who reports an allergy to penicillin with a rash and blistering of the skin (Stevens-Johnson Syndrome)?**	
Very Likely/Likely	373 (91.9%)
Neutral	3 (0.7%)
Very Unlikely/Unlikely	18 (4.4%)

* Where percents do not total 100%, the remaining percentage is a question not answered by respondent.

**Table 4:** Knowledge *.

Questions and Responses	N (%)
**How likely is a patient with a documented penicillin allergy truly allergic to penicillin based on skin testing/oral challenge?**	
Very Likely/Likely	174 (42.9%)
Neutral	28 (6.9%)
Very Unlikely/Unlikely	161 (39.7%)
**How likely is a patient to experience immune amnesia (e.g., “their body forgets their allergy”) after 10 years?**	
Very Likely/Likely	129 (31.8%)
Neutral	78 (19.2%)
Very Unlikely/Unlikely	75 (18.5%)
**What is the likelihood of cross-reactivity between penicillin and ceftriaxone (3rd generation cephalosporin)?**	
Mean% ± SD	36.2 ± 27.4
Median% (IQR)	29 (11–60.25)
**What is the likelihood of cross-reactivity between penicillin and cefazolin (1st generation cephalosporin)?**	
Mean% ± SD	51.6 ± 29.4
Median% (IQR)	50 (24–75)
**What is the likelihood of cross-reactivity between penicillin and carbapenems?**	
Mean% ± SD	34.4 ± 28.7
Median% (IQR)	26 (8–50.25)
**What is the likelihood of cross-reactivity between penicillin and sulfamethoxazole-trimethoprim?**	
Mean% ± SD	24.2 ± 25.9
Median% (IQR)	16 (0–40.75)

* Where percents do not total 100%, the remaining percentage is a question not answered by respondent.

## Data Availability

The data presented in this study are available upon request from the corresponding author.

## Supplementary Materials

The following supporting information can be downloaded at https://www.mdpi.com/article/10.3390/antibiotics15010094/s1.
