# Supplementary material for: Knowledge and Confidence of Physician Assistant Students in Managing Patients with a Documented Penicillin Allergy
Source: Antibiotics (Basel). 2026 Jan 16;15(1):94. doi: 10.3390/antibiotics15010094 (PMC12837476; doi:10.3390/antibiotics15010094)
Supplement: Supplementary file 1 [file antibiotics-15-00094-s001.zip › antibiotics-4078863-supplementary.pdf]

Supplemental Table S1: Class Comparison

Class Status	First-Year Students (Graduation Year 2022)	Upper-Level Students (Graduation Year ’20 – ’21)	P-Value
**Attitude**			
**How likely are you to avoid a beta-lactam antibiotic in a patient who reports an allergy to penicillin with nausea and vomiting?**			
Mean ± SD	2.4 ± 1.1	2.59 ± 1.1	
Median (IQR)	2 (2 – 3)	2 (2 – 4)	
**How likely are you to avoid a beta-lactam antibiotic in a patient who reports an allergy to penicillin with facial swelling?**			
Mean ± SD	1.4 ± 1.0	1.27 ± 0.8	
Median (IQR)	1 (1 – 1)	1 (1 – 1)	
**How likely are you to avoid a beta-lactam antibiotic in a patient who reports an allergy to penicillin with a rash and blistering of the skin (Stevens-Johnson Syndrome)?**			
Mean ± SD	1.3 ± 1.0	1.2 ± 0.7	
Median (IQR)	1 (1 – 1)	1 (1 – 1)	
**Confidence**			
**I am confident in my ability to assess/reconcile a patient’s documented penicillin allergy.**			
Mean ± SD	2.6 ± 1.1	2.1 ± 0.9	
Median (IQR)	2 (2 – 4)	2 (1 – 2)	
**I am confident in determining the drug of choice for patients with a documented penicillin allergy.**			
Mean ± SD	2.5 ± 1.2	2.0 ± 0.9	
Median (IQR)	2 (2 – 3.25)	2 (1 – 2)	
**I am confident in my knowledge of cross-reactivity among other antibiotics in patients with a documented penicillin allergy.**			
Mean ± SD	2.9 ± 1.4	2.4 ± 1.1	
Median (IQR)	3 (2 – 4)	2 (2 – 3)	
**I feel confident in my training during the PA program in managing patients with penicillin allergies.**			
Mean ± SD	2.2 ± 1.1	2.0 ± 1.0	
Median (IQR)	2 (1 – 3)	2 (1 – 2)	
**Knowledge**			
**How likely is a patient with a documented penicillin allergy truly allergic to penicillin based on skin testing/oral challenge?**			
Mean ± SD	2.9 ± 1.4	3.0 ± 1.4	
Median (IQR)	2 (2 – 4)	3 (2 – 4)	
Percent Correct	21%	43%	0.014
**How likely is a patient to experience immune amnesia (e.g. “their body forgets their allergy”) after 10 years?**			

Mean ± SD	2.8 ± 1.2	2.9 ± 1.0	
Median (IQR)	3 (2 – 4)	3 (2 – 4)	
Percent Correct	7%	3%	0.009
**What is the likelihood of cross-reactivity between penicillin and ceftriaxone (3rd generation cephalosporin)?**			
Mean ± SD	41.1 ± 28.7	31.9 ± 25.6	
Median (IQR)	35 (15 – 66)	25 (10 – 50)	
Percent Correct	11%	18%	0.261
**What is the likelihood of cross-reactivity between penicillin and cefazolin (1st generation cephalosporin)?**			
Mean ± SD	56.2 ± 28.2	47.8 ± 30.0	
Median (IQR)	60.5 (31.5 – 76.6)	50 (20 – 75)	
Percent Correct	2%	8%	0.045
**What is the likelihood of cross-reactivity between penicillin and carbapenems?**			
Mean ± SD	39.3 ± 28.7	30.3 ± 28.1	
Median (IQR)	34 (14 – 61.5)	23 (5 – 50)	
Percent Correct	14%	34%	0.003
**What is the likelihood of cross-reactivity between penicillin and sulfamethoxazole-trimethoprim?**			
Mean ± SD	29.5 ± 27.1	19.7 ± 24.1	
Median (IQR)	24 (5 – 50)	11 (0 – 28)	
Percent Correct	24%	52%	0.003

Supplemental Table S2: Confident vs. Other

Confidence Status	Confident (n = 289)	Not Confident(n = 107)	P-Value
**Attitude**			
**How likely are you to avoid a beta-lactam antibiotic in a patient who reports an allergy to penicillin with nausea and vomiting?**			
Mean ± SD	2.4 ± 1.1	2.4 ± 1.1	
Median (IQR)	2 (2 – 3)	2 (2 – 3)	
**How likely are you to avoid a beta-lactam antibiotic in a patient who reports an allergy to penicillin with facial swelling?**			
Mean ± SD	1.3 ± 0.7	1.5 ± 1.2	
Median (IQR)	1 (1 – 1)	1 (1 – 1)	
**How likely are you to avoid a beta-lactam antibiotic in a patient who reports an allergy to penicillin with a rash and blistering of the skin (Stevens-Johnson Syndrome)?**			
Mean ± SD	1.2 ± 0.7	1.39 ± 1.1	
Median (IQR)	1 (1 – 1)	1 (1 – 1)	
**Knowledge**			

**How likely is a patient with a documented penicillin allergy truly allergic to penicillin based on skin testing/oral challenge?**			
Mean ± SD	2.9 ± 1.4	3.1 ± 1.5	
Median (IQR)	3 (2 – 4)	2 (2 – 4)	
Percent Correct	26%	23%	0.273
**How likely is a patient to experience immune amnesia (e.g. "their body forgets their allergy") after 10 years?**			
Mean ± SD	2.8 ± 1.1	2.8 ± 1.1	
Median (IQR)	3 (2 – 4)	3 (2 – 4)	
Percent Correct	5%	5%	0.471
**What is the likelihood of cross-reactivity between penicillin and ceftriaxone (3rd generation cephalosporin)?**			
Mean ± SD	35.2 ± 0.8	38.9 ± 1.0	
Median (IQR)	26 (10 – 62.25)	32.5 (21.75 – 50)	
Percent Correct	13%	6%	0.006
**What is the likelihood of cross-reactivity between penicillin and cefazolin (1st generation cephalosporin)?**			
Mean ± SD	51.1 ± 0.6	53.0 ± 1.0	
Median (IQR)	50 (21 – 75.75)	50 (31.5 – 75)	
Percent Correct	5%	2%	0.072
**What is the likelihood of cross-reactivity between penicillin and carbapenems?**			
Mean ± SD	31.3 ± 0.8	43.2 ± 0.8	
Median (IQR)	24 (5 – 50)	35.5 (23.75 – 65.5)	
Percent Correct	23%	6%	< 0.001
**What is the likelihood of cross-reactivity between penicillin and sulfamethoxazole-trimethoprim?**			
Mean ± SD	20.3 ± 0.7	35.7 ± 1.1	
Median (IQR)	10 (0 – 28.25)	31 (14 – 50)	
Percent Correct	35%	14%	< 0.001

Supplemental Table S3:

Physician Assistant Program	Number of students enrolled during study period
University of South Carolina	60
Presbyterian College	64
North Greenville University	60
Campbell University	108
Charleston Southern University	60
University of North Carolina at Chapel Hill	40
Gardner Webb University	72
High Point University	70
Wake Forest University	176
Wingate University	110
Medical University of South Carolina	120
